# The Politics of Reproductive Signification: Navigating Legal Definitions of Parenthood in the Context of UK Informal Donor Conception

**DOI:** 10.1111/1467-9566.70277

**Published:** 2026-10-06

**Authors:** Leah Gilman, Alexus Davis

**Affiliations:** ^1^ School of Sociology and Criminology Manchester Metropolitan University Manchester UK; ^2^ Department of Sociology University of Manchester Manchester UK

## Abstract

Informal donor conception (IDC) has an ambiguous status under UK law. Whilst not illegal, it is discouraged by regulators and only partially recognised in attributions of legal parenthood. In this paper, we analyse IDC as a lens to explore the politics of reproductive signification. We use this term to refer to the power dynamics at play in deciding the meaning of reproductive processes in people's everyday lives. Drawing on interviews with 19 donors and 30 (intended) parents via IDC, we explore how they manage the relational significance of conception, gestation and care in the context of a legal regime which is often at odds with their intentions. Building on theories of legal consciousness and stratified reproduction, we demonstrate how the politics of reproductive signification encompass not only cis/heteronormative legislation but also the ways in which inequalities, relating to economic resources, racialised and classed parenting ideals and relationship status, shape possibilities to work with the law and resist its relational authority via practices of de‐animation or embrace legal ambiguity. Our analysis has implications for understanding how people negotiate the meaning of reproduction, particularly in the context of legal marginalisation.

## 1 Introduction

When our first daughter was born and we went to register her, they wouldn’t allow me to go on the birth certificate. Which absolutely broke my heart…But it made me feel better that it was obviously just my wife’s name that was going on there and nobody else’s.

(Amber)

Leah met Amber via video call as part of our research into informal donor conception (IDC) in the UK. She has three children: one teenager from a previous heterosexual relationship and two younger children with her wife, conceived via home insemination. Amber asked an old friend, Daniel, to be their sperm donor—a man she trusts and who shares her Black Caribbean/White European dual heritage. They have a good relationship but, as agreed, Daniel does not play a significant role in their family. Neither Daniel nor Amber's wife dispute that Amber is a mother to all three children. However, due to the complex positioning of IDC within UK law (detailed below), this is not reflected in her legal status and her name is unrecorded on her younger children's birth certificates—an absence which Amber feels keenly.

Amber's story, and those of other parents and donors in the study, raises questions about the *politics of reproductive signification.* We use this term to refer to the power dynamics involved in deciding what reproductive processes (broadly understood to include conception, gestation, feeding and childcare) mean. This includes consideration of whose desires and voices shape definitions of parenthood and how agency in such matters may be constrained or supported by institutional processes and access to resources.

IDC in the UK provides an interesting lens through which to explore the politics of reproductive signification due to its complex positioning in relation to legal and medical institutions. IDC refers to donor conceptions arranged outside of medical institutions. It is discouraged by the UK regulator which emphasises the health and legal risks (Gilman and Nordqvist 2023). Nevertheless, IDC has a long history in LGBTQ communities (Mamo 2007) and appears to be increasing in the UK, and other Global North contexts, facilitated by online ‘matching’ platforms and social media groups, which connect would‐be donors and recipients (Côté et al. 2025; Harper et al. 2017; Taylor‐Phillips et al. 2025) combined with financial barriers to clinical fertility treatment (F. Taylor et al. 2022).

IDC can provide a legally recognised path to parenthood and donation for some, but this is highly contingent. Under UK law, the person who gives birth is always the legal mother at birth. If they conceived via IDC and were (a) married or civil partnered at the time of conception, (b) conceived via artificial insemination *and* (c) the partner consented to this, *then* the partner will also be a legal parent, and the sperm donor will not. However, if any of these conditions are not met then the donor will be a legal parent, and any non‐biological co‐parent(s) will *not* be legally recognised at birth. After the birth of a child, non‐biological co‐parents can obtain legal parenthood via adoption (extinguishing the donor's legal parenthood) or apply for parental responsibility, conferring rights to make decisions about the care of their child(ren) without changing the legal parental status of the donor.

Applying this to Amber's circumstances, she is not recognised as a legal parent to her two youngest children because she and her wife (their birth mother) were not married until after their conceptions. At the time of interview, although not named on the birth certificate, Daniel was their legal father and Amber was applying for parental responsibility.

This article analyses in‐depth interviews with 30 (intended) parents^1^ and 19 donors via IDC, exploring how they attempt to manage the meaning of reproduction in this complex and somewhat de‐legitimised context. Building on theories of legal consciousness (Ewick and Silbey 1998; Harding 2008, 2010), and stratified reproduction (Colen 1995; Mamo and Alston‐Stepnitz 2015), we demonstrate how the politics of reproductive signification encompass not only cis/heteronormative legislation but also the ways in which broader inequalities, relating to economic resources, racialised and classed parenting ideals and relationship status, shape how such laws can be negotiated and the possibilities for resistance.

## 2 IDC in Context: Stratifications of (Queer) Reproduction

To begin, we situate IDC within shifting stratifications of (queer) reproduction. The concept of stratified reproduction captures the ways in which the reproduction of some is valued and supported, whilst others' is de‐valued and constrained (Colen 1995). The ability to reproduce and raise children is therefore structured by intersecting axes of oppression in relation to race/ethnicity, immigration status, socio‐economic class, disability and gender/sexuality (e.g., Bell 2010; Chen 2025; Mamo and Alston‐Stepnitz 2015; Smietana et al. 2018). Thus LGBTQ individuals often navigate assisted reproduction in the context of potential exclusion, misrecognition and stigmatisation (Leibetseder 2020; Nordqvist 2012a; Smietana and Twine 2022).Studies of queer reproduction particularly highlight the ways in which reproductive stratifications are ‘multilayered’ (Chen 2025), creating patterns of vulnerability and interdependencies, or reproductive bioprecarity, in relation to some groups more than others (Griffin and Leibetseder 2020). This is partly because accessing reproductive technologies often requires considerable economic, as well as social and emotional, resources in order to navigate medical treatment and exclusionary policies (Chen 2025; Leibetseder 2020; Mamo 2007; Smietana 2025; Y. Taylor 2009). For example, single people and LGBTQ couples frequently face additional barriers to publicly‐funded treatment due to heteronormative definitions of infertility (Bravo‐Moreno 2021; F. Taylor et al. 2022). Importantly, Leibetseder (2020) argues that cis/heteronormative legal and medical categories not only limit imaginaries and access to reproductive technologies but also create reproductive bioprecarity beyond the birth of a child via struggles for legal recognition.

Crucial for understanding IDC is a body of work which explores the relationship, between biomedicalisation and stratifications of (queer) reproduction (Bell 2010; Cai 2026; Mamo 2007; Mamo and Alston‐Stepnitz 2015). The growth of fertility medicine, and the accompanying biomedicalisation of (in)fertility, has expanded reproductive possibilities for some, including queer people (Mamo and Alston‐Stepnitz 2015), particularly in a context where private fertility clinics are often better resourced for performing queer inclusion than their public counterparts (Andreassen and Dahl 2026). However, this serves to further marginalise those without the resources to access it, or who remain under‐served or excluded from clinical routes (Bell 2010; Cai 2026; Mamo and Alston‐Stepnitz 2015). It is important to note that biomedicalisation has a moral dimension to it. Clinical routes are frequently positioned as the only safe, ethical and legitimate way to access assisted conception (Nordqvist 2011; Taylor‐Phillips et al. 2025) and more medicalised pathways (i.e., IVF rather than IUI) are associated with neoliberal imperatives to optimise and manage one's own fertility (Cai 2026).

Our findings, and those of previous studies with IDC users (Côté et al. 2025; Taylor‐Phillips et al. 2025) support arguments that biomedicalisation and commercialisation of (in)fertility heighten stratifications of reproduction in relation to class and race, particularly as they intersect with gender and sexuality (Bell 2010; Mamo and Alston‐Stepnitz 2015; Tam et al. 2025). Previous studies demonstrate that the decision to use IDC is often motivated (at least in part) by financial barriers to clinical routes or experiences of marginalisation in clinics (Côté et al. 2025; Jadva et al. 2018; F. Taylor et al. 2022). However, access is not our primary focus here. Building on Leibetseder's (2020) consideration of the relationship between cis/heteronormative laws and reproductive bioprecarity, we are concerned with how the ability to attribute meaning to reproductive processes is also stratified. We focus on inequalities *between* IDC users, examining how positionality and resources shape the options available for negotiating reproductive signification.

## 3 Reproductive Signification Beyond the Genetic‐Nuclear Family

Our study thus also contributes to an expanding body of social scientific scholarship which explores how people navigate kinship in the context of assisted reproduction (Edwards and Salazar 2009; Franklin 2001; Hargreaves 2006; Thompson 2005; Keaney 2023). These studies collectively demonstrate that the social significance of different reproductive processes, including genetic inheritance, gestation, feeding and care, is highly contingent. Depending on whether donor gametes and/or surrogacy are used, varied reproductive processes will be emphasised or de‐emphasised as relevant to parenthood and what is counted as ‘biological’ can also vary according to kinship intentions, often intertwined with categorisations of race and ethnicity (see e.g. Bestard 2009; Franklin 2001; Keaney 2023; Thompson 2005). Typically, when parenthood is defined in the context of donor conception, intended parents and clinicians attempt to ‘screen out’ the donor's significance as a genetic parent, whilst ‘biological’ connections fostered via gestation and breastfeeding, as well as the importance of everyday care, will be emphasised (Nordqvist 2012b; Ryan‐Flood 2009; Thompson 2005).

However, studies of lesbian and solo parents also demonstrate that the status of non‐biological parents does not *necessarily* rely on the exclusion of the sperm donor from a socially significant role. Research shows that some intended parents predominantly queer and/or solo parents, choose known donors, often because they view contact with the donor as important to their children (Almack 2006; Haimes and Weiner 2000; Hertz 2002; Ryan‐Flood 2009). Known sperm donors may be attributed, and come to play, a range of roles in relation to solo and lesbian mother families and their children (Almack 2006; Dempsey 2010; Haimes and Weiner 2000; Hertz 2002; Ravn 2021; Ryan‐Flood 2009; Y. Taylor 2009).

Such research could be interpreted as evidence of individual agency regarding how reproductive processes are understood, with the meaning of genetic or biological processes being made to matter (or not) in line with individuals' wishes. However, Thompson's (2005) analysis shows that the making of parents and non‐parents via fertility treatment requires ‘choreographies’ of legal, social, technical, financial and medical practices. Appointments must be coordinated, gametes selected, stored and labelled, the right forms signed at the right times, in order for a particular baby to be recognised as the child of its intended parents only. It is a complex process involving multiple actors acting with common purpose.

As Nordqvist (2011) notes, the clinic acts as a ‘container’ for such choreographies, managing the legal and social complexities arising with donor conception. In this paper, we build on Nordqvist's work by examining how people manage the meaning of reproductive processes in a context where the clinic (and the legal statuses this affords) cannot be relied on to ‘choreograph’ their significance as intended. Instead, legal framings may actively work against the meanings which parents, donors, and potentially donor conceived people, wish to attribute to reproductive processes.

## 4 Legal Consciousness

To examine how participants navigate this complex legal positioning, we drawn on Ewick and Silbey's (1998) theory of legal consciousness, particularly as extended by socio‐legal scholars of queer kinship. Legal consciousness research examines how people relate to legal institutions and processes in the context of their everyday lives and examines how this is shaped by power and inequalities. Ewick and Silbey’s (1998) typology sets our three different forms of legal consciousness: (1) ‘before the law’, in which the law is understood as an independent, immutable authority beyond the control of individuals; (2) ‘with the law’, in which the law is something to be navigated strategically in pursuit of one's desired aims and (3) ‘against the law’, in which the law is something to be avoided, resisted or evaded.

A number of scholars have applied theories of legal consciousness in the context of queer kinship (see Hull 2016). These studies show how people exhibit varied and multiple forms of legal consciousness to navigate queer kinship in the context of different legal regimes (Baumle and Compton 2018; Hull 2016). Examining same sex marriage and second‐parent adoption, respectively, Hull (2003) and Connolly (2002) demonstrate how same‐sex couples may simultaneously value legal recognition for both practical and symbolic reasons, at the same time as seeing ‘the law’ as something to be resisted for its heteronormative logic. Similar tensions are evidenced in Harding's (2008) analysis of lesbian and gay men's attitudes to the introduction of civil partnerships in the UK; legal recognition can represent practical and symbolic equality, and simultaneously, new forms of inequality for those who remain excluded or disadvantaged (see also Shipman and Smart 2007).

Harding (2010) extends conceptualisations of resistance to the law. She finds few instances of the evasive resistance described by Ewick and Silbey (1998). Instead, she identifies forms of ‘stabilising resistance’, describing how LGBTQ people, simply by visibly existing, provide resistance to heteronormative laws. Second, she describes ‘moderating resistance’—attempts to challenge or contain the negative impact of discriminatory laws and institutions, for example via Pride marches. Third, least commonly, she describes ‘fracturing’ forms of resistance which involve more direct attempts to challenge or change the law. We extend Harding's (2010) framework, focusing on the how positionality and resources shape the forms of resistance which are possible in the context of IDC.

## 5 Methods

The data we share in this paper were produced as part of the Digital Donor Conception research study funded by The Wellcome Trust. Ethical approval was granted by The University of Sheffield and Manchester Metropolitan University. The study explores experiences and perceptions of IDC, as well as the digital platforms which facilitate these. In this article, we focus on findings from in‐depth interviews conducted with (intended) parents (*n* = 30) and sperm donors (*n* = 19) which explored people's experiences of donating, conceiving or trying to conceive via IDC.

All interview participants contacted us after seeing one of our advertisements. These invited participation from people who (a) considered themselves a parent/intended parent of a donor conceived child or a sperm donor, (b) had arranged conception/donation ‘informally’, defined as outside of a fertility clinic or sperm bank and (c) had a connection to the UK. We shared these adverts via UK‐based support organisations for donor conceived, LGBTQ and solo parent families, people experiencing infertility and via platforms used for IDC. All participants were offered a £20 gift voucher in recognition of their time and contribution.

We received expressions of interest from 223 people—more than we had resources to interview and so implemented a selection process. This involved monitoring the diversity of our sample in relation to categories of interest (e.g., sexuality, relationship status, ethnicity and children's ages) and prioritising contacting potential participants whose circumstances or identities were less represented. We also sought to screen out participants who we strongly suspected did not meet our inclusion criteria (so‐called ‘imposter participants’ (Santinele Martino et al. 2024)). If we judged that the information which a potential participant shared was highly contradictory or described IDC happening in ways which we understood to be impossible in the UK, we requested more information, and if these remained unexplained, did not invite them for interview. For the same reasons, data from two interviews were excluded from this analysis. As Garcia‐Iglesias et al. (2025) recognise, such exclusions relied on our prior knowledge. This did not include direct experience of IDC but was shaped by Leah's previous research on donor conception and our experiences as White British (Leah) and Black US‐American (Alexus) women living in the UK. There was therefore a risk of excluding narratives which undermined our expectations. However, we concluded we had an ethical obligation to limit the impact of consciously fabricated narratives of IDC in our analysis and sought to mitigate this risk by regularly reflecting on and questioning our own decisions.

Table 1 details the sample of (intended) parents and donors included in this analysis. It is diverse in relation to participants' sexuality, relationship status, age and household income, the ages of any children and timings of donations. However, there is more limited variation in relation to ethnicity and education; 78% of participants identified as White and 86% were educated to degree level. Furthermore, all (intended) parents had used, or were considering, IDC in the context of a LGBTQ partnerships (including same‐sex female and *trans*‐male/*cis*‐female couples) or as solo parents. Nineteen (intended) parents and eighteen donors had engaged seriously with specialised digital platforms (e.g., Facebook groups or Pride Angel) for donating or seeking sperm. Fifteen (intended) parents and five donors had found a donor/recipient via pre‐existing relationships and networks.

**TABLE 1:** Sample demographics and circumstances.

		(Intended) parents	Donors
Gender	*Cis*‐woman	26	0
	*Cis*‐man	0	19
	*Trans*‐man	3	0
	“Other” (excluding non‐binary)	1	0
Ethnicity	White British/Irish	21	14
	White other	2	1
	Black British/African/Caribbean	2	2
	Asian/Asian British	1	0
	Mixed/Multiple	3	2
	Missing	1	0
Sexuality	Gay/lesbian	15	4
	Bisexual	4	1
	Heterosexual	8	14
	Greysexual	1	0
	Missing	2	0
Relationship status	Single	13	8
	Married or civil partnered	9	5
	Living with partner	1	2
	In relationship (living apart)	5	0
	Divorced/Separated	0	2
	Widowed	1	2
	Missing	1	0
Age (at interview)	21–30	3	1
	31–40	15	8
	41–50	9	2
	51–60	0	4
	61–70	1	4
	Missing	2	0
Disability or long‐term health condition	Yes	7	3
	No	21	16
	Missing	2	0
Household income	£0–24,999	6	1
	£25,000–49,999	12	6
	£50,000–74,999	3	3
	£75,000–99,999	6	1
	£100,000+	1	7
	Missing	2	1
Highest educational qualification	GCSE or equivalent	1	0
	A‐level or equivalent	2	2
	Undergraduate degree	10	8
	Postgraduate qualification	16	8
	Missing	1	1
Years in which donated	1995–1999		1
	2000–2009		1
	2010–2019		10
	2020–2024		14
	Missing		2
Ages of any children^a^(parents only)	Under 5	14	
	5–11	10	
	12–17	4	
	18+	1	
	Pregnant	2	
	None (trying to conceive)	4	
	Missing	1	

^a^ In some cases, this includes children who were not conceived via informal donor conception.

Interviews took place during 2024 and lasted between 45 minutes and 2.5 hours. They were conducted via video call or in‐person, in a café, pub or participant's home. Most (43) participants took part in one‐to‐one interviews with either Leah or Alexus, and the other six choosing to take part in a joint interview, with either their partner or donor/recipient. Participants were asked about their journey to IDC, how IDC was organised and their experiences of IDC in the short and long‐term. Participants were encouraged to demonstrate how they had engaged with digital platforms in the process.

All interviews were audio recorded, transcribed verbatim and anonymised via pseudonymisation and obscuring identifying details. To address the particular sensitivities of identifiability in IDC, all participants were offered the option to review and redact their anonymised transcript. We then used Nvivo 12 to code the transcripts using a mixture of descriptive and analytic codes, initially both coding the same transcripts to promote inter‐coder consistency.

For this paper, we undertook multiple readings of the data coded as ‘legal considerations’ and/or ‘kinship,’ with a view to understanding how different participants managed the meaning of reproductive processes in relation to the law and how this was shaped by positionality and resources. During this process, Ewick and Silbey's (1998) and Harding's (2010) typologies of legal consciousness and resistance provided heuristics to think with, but as others have observed, we found that multiple forms of consciousness were often evident even within short extracts from interviews. Hence, we do not use these categories as an overarching structure to our analysis. Instead, we first explore the ways in which participants positioned the law as relationally significant, before focusing on ways it could be positioned as irrelevant or its ambiguity embraced.

## 6 Positioning the Law as Relationally Significant

(Intended) parents and donors in this study had often spent considerable time researching the legal context to IDC. They had read posts and asked questions in LGBTQ family and sperm donation social media groups and browsed government and fertility lawyers' websites. Occasionally, they discovered misleading or unclear information but the vast majority reported accurate, sometimes extremely detailed, understandings of relevant laws. Although we should acknowledge most were degree‐educated, we observed no relationship here with formal educational qualifications.

Many used this knowledge to seek legal recognition for their families. The clearest example of this was couples who married or civil partnered in order to secure legal parenthood for a non‐biological parent:

Innes So we got civil partnered in [early 2020s], and 90% of the reason of doing that was to make legal parental stuff easier

Harry Yeah, I don't think we would've done it otherwise. It wasn't something we were particularly invested in. (Harry and Innes, intended parents)

I want [partner] to have the same legal rights from the beginning. If I get home and I'm crazy and decide this isn't your child, I want someone to be stood there legally going, ‘That's not true’.

(Britney, parent)

Britney, Harry and Innes all exhibit elements of ‘before the law’ legal consciousness (Ewick and Silbey 1998). Their decisions to formalise partnerships are guided by an understanding of ‘the law’ as an authoritative power which can fix the meaning of reproductive processes. Britney recognises that the meaning of reproductive processes is contingent (Thompson 2005) but seeks to use the law to fix a singular truth, even if that were to go against her own future wishes. However, this is a power which she strategically chose to activate and as such it also resonates with Ewick and Silbey's (1998) descriptions of ‘with the law’ consciousness.

However, not all participants had the option to use marriage or civil partnership to manage the legal status of the donor. Daniella, a solo mother with three children (two via IDC) described how not having ‘someone else to put in the second parent slot’ (referring to the birth registration process) made her legally vulnerable. Charlie, also a solo mother, described her experience:

Stressful, unpredictable, anxiety‐inducing, I’m sure many of the things that you would still have if I went down the more formal route. But I guess more—it just feels like you haven’t got any legal protection, you know?

(Charlie, parent)

Charlie's reference to ‘legal protection’ combined with a despairing tone resonate with ‘before the law’ consciousness; here, the law is an authoritative power which she wants to access but cannot. Charlie's anxiety was compounded by a low income, commenting that she ‘just had to hope’ that her donor would not pursue contact in the future because ‘she couldn't afford a proper legal –’. Her sentence tails off but presumably she is referring to lack of funds for a solicitor or court costs. Thus, the ability to manage reproductive signification with the law was constrained by both class and relationship status.

The ability to use marriage/civil partnership as a route to legal parenthood was also stratified in more subtle ways related to time. There is a heteronormative logic to the principle that legal partners are recognised as co‐parents, only if the partnership is registered prior to conception, suggesting that a ‘serious’ relationship should pre‐date trying to conceive. Many LGBTQ individuals do value such a timeline (see Pralat 2021), but for some participants, this temporal logic did not apply. Ria and Tara explained that they had already been trying to conceive when they first met their partners. As women who both, to different degrees, expressed alignment with queer politics (e.g., Warner 2000) and critiqued institutionalised heteronormativity, pursuing motherhood was not something which they considered dependent on their relationship status and so these two life projects (coupledom and parenthood) existed for a time in parallel before becoming intertwined. By the time their children were born, their partners had become co‐parents—a social status which evolved during pregnancy rather than being entirely pre‐planned. According to law, reproductive signification is not expected to evolve in this way, at least not for queer families (contrast the English idiom of a ‘shotgun wedding’ in which a heterosexual couple quickly marry after an unplanned pregnancy). Consequently, both Ria and Tara's partners do not have legal parenthood. These participants, and several others, described it as unjust that a birthing parent could, as Amber put it, ‘take any man off the street’ to the registry office and name them as their child's father but not their female partner. Whilst this scenario would be fraud, it is plausible and Amber highlights the inequalities in who is able to work ‘with the law’ (cf. Ewick and Silbey 1998) in this way.

Another way in which people strategised ‘with the law’ was through anticipation of court cases. None of the interview participants had been involved in a formal legal dispute related to IDC but stories and imaginaries of the family courts shaped practice. A clear example of this is the creation and signing of written ‘donor agreements’—often based on templates shared in online communities—which outlined the respective roles of donors and recipients in relation to any child conceived. Agreements frequently used legalistic language (such as ‘parental rights’, ‘financial responsibilities’ and ‘custody’) and formats, including the use of numbered clauses, definitions of terms and were signed and dated. One parent described how she and her wife had asked their neighbours to formally witness their signatures.

Many participants referred to hypothetical court proceedings to explain the making of donor agreements:

I did find a sperm donor’s agreement, like the known sperm donor’s agreement, just to make sure that not only I’m protected, but also she’s protected as well. It’s not exactly legally‐binding, but if anything does arise, then that could be held in court.

(Kai, donor)

Whilst valued, these agreements were often described explicitly as ‘not legally binding’ (Zira, parent) or as having ‘no legal weight’ (Will, donor). This was the stated reason that very few involved a solicitor.

In a more unusual form of preparation for the courts, Amber had created a record of her parenting in anticipation that she might one day need such evidence in court:

Amber So, in our relationship I'm the one that takes care of basically anything paperwork‐wise and all the rest of it. So, with the kids going to school, for example, going to nursery, I've always been the one to fill out those forms…I've purposely signed those forms because if it came to it and I needed to go to court I can use that as evidence. I support my children financially. I'm the one the pays their nursery bill, all the rest of it. So I purposely make all of that contact

Leah So you've made a paper trail

Amber Yeah. Exactly. Yeah, and in court they ask for evidence like that as proof that you've financially provided for your children and all the rest of it. So I've got it all. So they can come for me if they want to

It is not entirely clear who ‘they’ refers to in this last sentence. However, Amber's strong relationship with her donor and wife suggest she is referring to the family courts or broader legal system. Reflecting patterns observed in relation to same‐sex marriage and second parent adoption (Connolly 2002; Hull 2003), her systematic record keeping combines ‘against’ and ‘with’ the law consciousness (Ewick and Silbey 1998). Amber is strategising in anticipation of interactions with legal institutions which she envisages may fail to recognise, if not be actively hostile towards, her. Here it is relevant that Amber is not only in a same‐sex relationship but also someone who has a child from a previous relationship, is from a racialised minority, and although she is a graduate with a professional job, she has a household income below the UK average. Given the classed and racialised dimensions of reproductive and parenting ideals (Hamilton 2020; Y. Taylor 2009; McCormack 2005), institutional racism within the UK justice system (Monteith et al. 2022) and the costs of solicitors, Amber perhaps has particular reason to anticipate marginalisation by the family courts.

Three participants also sought to manage reproductive signification post‐birth via adoption or applying for parental responsibility. All encountered obstacles. Zira (a parent via surrogacy and IDC) and Amber, who were seeking adoption and parental responsibility respectively, expressed frustration that social workers and family court staff seemed unprepared to support families similar to theirs. They both reported that legal processes, such as forms and online information, seemed designed for heterosexual parents following separation. In addition, James, a trans birthing father, described how he and his partner, Sara, delayed pursuing parental responsibility for Sara because this required a birth certificate; they had delayed registering their child's birth in the hope that Freddy McConnell would win his high‐profile case against The Registrar General for England and Wales in which he sought the right to be registered as his child's father or parent:

Currently trans men who give birth have to be listed as mother, there’s an oncoming court case around that and we were hoping …. I wouldn’t have to go down as mother. So, actually we delayed getting [child’s]’s birth certificate basically until he was one day short of a year old…That added further complication in that we couldn’t go down any pathway to get [partner’s name] parental responsibility without having the birth certificate. So, we were in limbo in that aspect for a year.

(James, parent)

For James and Sara, ‘fracturing’ resistance to one aspect of the law (the gendering of birth registration), limits their ability to work ‘with’ the law in relation to legal parenthood and parental responsibility, because the two processes are so intertwined. This is in addition to the obstacles which trans parents encountered when negotiating *cis*‐normative legal frameworks where it was often unclear how the gendered language of the law would apply to them. As James put it, as a trans parent, ‘you've got be quite on top of it.’ Thus reproductive signification is stratified for trans parents both by cisnormative laws *and* the additional social and financial resources required to figure out how these apply and if/how they can be worked with or resisted.

## 7 Positioning the Law as a Relational Technicality

Whilst usually working ‘with the law’ where possible, people also managed reproductive signification through, what we term, ‘de‐animation’ of the law. De‐animation describes practical and discursive acts through which participants positioned legal definitions as ‘technicalities’ with limited significance to their lived relationships. Engaging in such processes enabled participants to see the law as, whilst not irrelevant in general terms or even necessarily unjust, unlikely to ‘come to life’ when applied to their specific circumstances.

De‐animation is evident in the ways donors and (intended) parents built and referenced trust. Many participants explained that, whilst they were aware that ‘technically’ their position was legally risky, they had no concerns because they had complete trust in their particular donor or recipient(s) not to undermine their agreement:

I mean obviously there are, if I didn’t know him so well, I might be worried that he would take advantage of the situation or he might try to get custody of her or he might start demanding things of me, but I know 100%, I trust 100% what he wants and that is not what is going to happen.

(Chris, parent)

Stuart I do know, whether they know it or not, that strictly speaking, that child could be entitled to financial maintenance from me

Leah How much of a risk do you feel it is?

Stuart Zero. I don't think any of the women that I'm working with would actually do that. (Stuart, donor)

We can interpret Stuart and Chris' words as a form of ‘moderating resistance’ to the law (Harding 2010). Although the discrepancy between legal definitions of parenthood and their own is acknowledged, developing trusting relationships enables them to position this as unapplicable to their own circumstances.

In addition to inter‐personal trust, participants also described trust in their situation. This is what Nordqvist (2011) terms the ‘economy of trust’ in which both parties had a vested interest in maintaining the donation agreement. Leo, a sperm donor, explained it as follows:

I am not too worried about people putting in claims for financial reasons because they are equally worried about me wanting to get in contact [laughs]. So, it is kind of a mutual truce. Yes, we both don’t want to go down that route.

(Leo, donor)

The economy of trust could be strengthened through either purposefully selecting, or reflecting on the choice of, a particular donor or recipient. So, whilst there was widespread concern about ‘prolific’ donors, participants occasionally commented that donors who had donated multiple times were less likely to ‘use’ their legal parental status to pursue parental responsibility and this could be appealing:

And then if the donor has more children from more women, it’s [sic] much less likely to come after you. …I see the issue if the man doesn’t have kids, or maybe has one, so then they might want to jump in and parent.

(Ada, parent)

For donors too, legal definitions of their role could be de‐animated through the selection of recipients. Leo, for example, described his recipients as ‘well off’ and so ‘less likely….to break an agreement.’ Some donors therefore preferred to donate to those with a level of financial security and/or who they perceived to be in a ‘stable’ partnership (relationship breakdown being seen as a financial risk). Thus, in the context of high demand for donors, definitions of ‘ideal’ recipients and donors' preferences can reproduce similar reproductive stratifications related to class and relationship status as shape access to clinical donor conception (Mamo and Alston‐Stepnitz 2015; Thompson 2005).

De‐animation could also involve arranging IDC in ways which made it *practically* challenging for the other party to disrupt the donation agreement. In a number of cases, donors and recipients had withheld, or reported others withholding, identifying information about themselves as a way to mitigate legal risk (see also Gilman and Davis 2026). Chris, for example, used an alias with his donor. Ada had carefully avoided sharing her address with her donors. Some donors used a ‘donor profile’ and/or ‘donor email’ for the purposes of communicating with recipients. Eloise, who has tried to conceive via IDC explicitly linked anonymous practices with donors' precarious legal position:

You don’t want donors to be subjected to maintenance payments. That’s their biggest fear and that encourages them to be anonymous which as we know is a bad thing. So perhaps if they knew that they were protected they’d be much more open to be being open and honest about their identity, meeting with families if they’ve got them.

(Eloise, parent)

It should be noted that withholding of identifying information was not necessarily absolute (e.g., participants sometimes shared their name but not their address) and did not necessarily mean there was no ongoing contact. Many donors who used a pseudonym remained in contact with parents after the birth of a child. However, by limiting the disclosure of identifying information, both parties maintain the possibility of disconnection and thus de‐animation of the law.

It is worth pausing here to emphasise that de‐animation was not a denial of the law's power, in general terms, but a practice of actively creating or referencing circumstances in which this power is de‐activated. Thus, it often sits alongside ‘before the law’ legal consciousness. Although some, such as Stuart and Chris above, presented de‐animation as long‐term and secure, others were mindful that, should circumstances change, the law's power to define the meaning of reproduction could be re‐activated.

Caroline's experience provides some insight here. She has an adult donor conceived son and explained she had initially opted for no contact with her donor:

Well, because of the legal thing, I thought we wouldn't really have any contact with him. Because partly, I was like, scared, you know, what, if he applied for custody, not that I didn't trust him, but because of the legal situation.

Later, she continued:

It was just kind of that anxiety, and also myself being a single parent, I thought, well, if I'm made redundant and I have to sign on and I kind of created a fictional, semi‐fictional backstory in case of that. Yeah, it was a worry that, because in English law, you can't, you're not really allowed to…

(Caroline, parent)

As a solo mother, Caroline was concerned that, should her financial circumstances change, the donor's legal status could be activated as relationally significant. Hence, she layers strategies of de‐animation, relying not only on trust but also declining any contact.

However, Caroline's position on contact softened over time and, during her child's childhood, she moved from ‘letter box’ contact to regular meetings with the donor. Such relaxations were common. This is partly a consequence of family relationships becoming more established and parents imagining that, should a legal dispute arise, it is increasingly unlikely that a judge would see any disruption to the de facto family as being in their child's ‘best interests’. As Mary, another parent explained, now that their child is 6 years old, there is ‘no way’ their donor would secure ‘50/50 custody’. In addition, the passing of time could strengthen trust between donors and recipients. Rebecca explained that initially she and her ex‐partner had not shared their address with their donor, leaving open the possibility of cutting contact. Some years later, this no longer seemed necessary:

It worried me that if we can’t get away from him so—but once we’d moved to where I still am now, we knew that he was good by then.

(Rebecca, parent)

The passing of time cemented intended reproductive significations through their repeated enaction and thus further de‐animates the ‘letter of the law.’

## 8 Embracing Ambiguity as Resistance

In the above examples, whether working with or seeking to de‐animate the law, participants acted to ensure that the meaning attributed to reproductive processes aligned with their own pre‐defined intentions. In contrast, some participants resisted the idea that reproductive signification could, or should, be fixed or codified. Instead they celebrated, albeit cautiously and partially, the idea that the meaning of reproduction can be ambiguous and evolve over time. Here, what regulators present as legal risks (Gilman and Nordqvist 2023) are re‐framed as flexibility, often discussed in relation to a child's emerging desires and relationships.

Lucy's story is illustrative. She is a solo mother who conceived with the help of a sperm donor she initially met online:

Lucy I mean I understand legally he's considered my child's father, if he was to choose to go down the court process and seek parental responsibility. But ultimately, biologically, he is my child's father, so that's a fact, I was fairly confident he wasn't going to be seeking parental responsibility. I was fairly confident that I was taking a balanced risk with that and for me I was comfortable with it. Ultimately, if he had, he is—my child is half biologically his, so I would have worked out a way to navigate that

Alexus And how do you feel about that legal context[….]?

Lucy I think it's a shame that, sort of, written agreements between recipient parents and donors have no weight in court. But equally I think, because I do a lot of work with the family court, I know they will always make decisions in the best interests of the child. And that if a child has a biological parent who wants to be part of their life, the court will ordinarily view that as a positive. And, actually, I do recognise there are positives to that. Would I like to see informal donor conception have the same protections as clinic donations? Yes, but do I believe that donations through a clinic should only be open ID at 18? No. So, I think, yeah, I think sometimes parents' desire to be parents can override the donor‐conceived child's views and I think that's what the court is there to make decisions about. So, I think I see it from both sides, really

We quote Lucy's interview at length to demonstrate the ambivalence and complexity in her relationship to the law. She critiques the lack of legal recognition for her family and donation agreement. However, for Lucy, the solution is not simply for laws around IDC to be changed to mirror clinical donor conception because, for her, a system where donors are anonymous until eighteen and have no status at all in relation to donor conceived children is also problematic. Thus, the kinds of legal changes Lucy is advocating for are not ones in which the meaning of reproduction is entirely fixed pre‐conception. Instead, she places a value on reproductive signification being, to a certain extent, open‐ended. A large part of her rationale for this is the moral value she places on taking account of children's developing views (see also Almack 2006; Donovan and Wilson 2008).

This open‐ended approach to the donor's role was echoed by several parent participants. Penny explained why she and her wife did not involve a solicitor in their agreement with their donor:

It might have been a thought, and then we never did it. I suppose ultimately, if he wanted contact, he could have it. I suppose I wouldn’t necessarily object to be honest. Like I said, it was one of the things that I wanted anyway.

(Penny, parent)

After explaining that she trusted her donor not to ‘claim access,’ Tara said:

I know even if that happens with [donor], like [son’s] at an age now where I think, you know, we could cope with that and [donor’s] a nice guy. At the end of the day a child can’t have too many people that love them. So, I don’t think it was something I was ever majorly worried about.

(Tara, parent)

For both Penny and Tara, the donor seeking further contact was something they envisaged could be added into their existing family structures. In these examples, the legitimacy of their families is not dependent on the complete exclusion of the donor from any parental or parent‐like role. In this sense, as in Harding's (2010) formulation of stabilising resistance, these participants resist the heteronormative two‐parent logic embedded in UK law by enacting their family lives in an alternative manner.

However, to return to Lucy's interview, we can see that there is another fundamental principle of family law which she clearly does value—the prominence given to the ‘best interests of the child’. In relation to this principle, she exhibits ‘before the law’ legal consciousness, submitting herself to the benign authority of the courts to make such judgements. It is relevant here that Lucy is white, highly‐educated and has professional experience of the family courts all of which arguably give rise to expectations of positive outcomes should she find herself in a position where her parenting is subject to state judgement. In addition, as was also the case for Tara and Penny, she is speaking as a birth parent, therefore her own status as a legal parent is secure. Thus, we suggest that these participants' ability to take an open‐ended approach to reproductive signification and, in doing so, to resist some of the heteronormative assumptions of the law, was contingent on the security of their own status as birth mothers as well as an expectation that their voices will be accorded significant weight should any legal dispute arise. There is a stark contrast with Amber's words—with which we began this paper—wherein the donor's exclusion from the birth certificate ‘made [her] feel better’ in a context where she has no legal status in relation to her two youngest children.

## 9 Conclusion

In this article, we have discussed how people manage the social significance of reproductive processes in IDC, a context where medical and legal institutions cannot necessarily be relied on to ‘contain’ (Nordqvist 2011) or ‘choreograph’ (Thompson 2005) this in line with parents' and donors' intended or lived relationships. The complex and precarious positioning of IDC in relation to UK law has enabled us to explore, what we refer to as, the politics of reproductive signification—the power dynamics which shape how shared meanings of reproductive processes (conception, birth and everyday care) are established.

We propose the politics of reproductive signification as an important, but currently under‐investigated, dimension of stratified reproduction. Our analysis highlights that reproduction is stratified not only in the ways which reproduction is variably supported and valued for different groups, but also in how power to decide what reproductive processes *mean* is distributed in unequal ways. These stratifications shape whose reproductive intentions or desires are recorded, codified and acknowledged as relationally relevant and whose are side‐lined or rendered precarious. The politics of reproductive signification means that, even when access is possible, some people engage in reproduction and family‐building under the threat of invisibilisation or misrepresentation, whilst others feel secure in the knowledge that their reproductive intentions will be honoured.

Our findings show that the law plays an important but not determining role in the politics of reproductive signification Laws which assign particular meanings to reproductive practices are negotiated and resisted in varied ways and unequal contexts. In our study, (intended) parents and donors often worked ‘with the law,’ where possible, to ensure attributions of parenthood and potential judicial decisions, aligned with their intentions. However, the relational significance of the law was also contested. Building on Harding's (2010) ‘moderating resistance,’ we introduce the concept of de‐animation to describe practical and discursive acts via which people sought to position the law as a ‘technicality’ with minimal bearing on their everyday lives and relationships. In addition, a minority of participants embraced legal ambiguity. In doing so, they resisted the idea, embedded in UK definitions of legal parenthood, that the meaning of reproductive processes could, or should, be fixed pre‐conception. Importantly, our analysis shows that these different approaches were not equally available to all. In line with Leibetseder (2020), we found some were rendered particularly precarious by the categories of cis/heteronormative legislation which limited possibilities to work ‘with’ the law (e.g., solo and trans parents), others by the ways in which broader inequalities shaped how such laws could be negotiated and resisted. In particular, the ability to embrace ambiguity in relation to reproductive signification perhaps relied on a level of legal security for one's own parental status and an expectation of positive recognition of one's parenting ‘before the law’. Those with fewer options or resources were more likely to rely on forms of de‐animation which often raised ethical concerns for participants, for example withholding identifying information or selecting a donor who had donated to multiple people.

We hope that future research will explore the politics of reproductive signification in other contexts, particularly those in which lived and intended meanings of reproduction are ambiguous or liable to misrepresentation or contestation, both legally and socially. Such contexts are not limited to those involving assisted reproduction and could include multi‐parenting, platonic co‐parenting and post‐separation childcare arrangements as well as surrogacy and cross border reproductive care. Bringing together theories of legal consciousness and stratified reproduction allows us to examine the complex relationship between inequalities, the law and reproductive meaning‐making. We suggest that such work requires attention to the processes of de‐animation and embracing ambiguity which we have identified in this paper and which are likely to have broader relevance for understanding how people manage disjuncture between (inevitably somewhat messy and fluid) lived relationships and the ways these are codified in law.

Our research points to the need for legal reform in order to better recognise and support the diverse ways in which families are formed over time and reproductive processes are inscribed with varied and contingent relational significance. In the case of IDC, this might include: extending routes for solo legal parenthood (currently only possible, in the UK, via clinics); addressing barriers to legal parenthood for trans parents; affording some legal status to pre‐birth donor agreements; reforming birth registration to allow for more than two legal parents to be recorded and training social workers, court officials and registrars to ensure that information regarding legal processes is inclusive of diverse family forms. Improving legal routes for managing reproductive signification would likely discourage reliance on some of the forms of de‐animation which participants themselves often found uncomfortable (e.g., withholding identifying information) and which have potentially negative consequences for the donor conceived people who are born as a result. Furthermore, whilst many participants in this study did seek and value legal recognition for defined parent and donor roles in IDC, care is needed to ensure that legal clarity is not imposed in ways which limit open‐ended reproductive signification, which can be responsive to children's developing understandings of kinship, or impose heteronormative two‐parent models of family where this is not reflective of people's lived experience and familial intentions.

## Author Contributions

**Leah Gilman:** conceptualization, investigation, funding acquisition, writing – original draft, writing – review and editing, formal analysis. **Alexus Davis:** writing – review and editing, investigation, formal analysis.

## Funding

The research reported in this article was funded by The Wellcome Trust [Grant No. 225536/Z/22/Z].

## Ethics Statement

This study was granted ethical approval by the University of Sheffield Sociological Studies Ethics Committee (Reference No. 053411) and the Arts and Humanities Research Ethics and Governance Committee at Manchester Metropolitan University (Reference No. 75019).

## Consent

The authors have nothing to report.

## Conflicts of Interest

The authors declare no conflicts of interest.

## Permission to Reproduce Material From Other Sources

The authors have nothing to report.

## Data Availability

Data supporting this publication will be made available on Manchester Metropolitan's research repository (e‐space) by April 2027: https://e‐space.mmu.ac.uk/. In order to protect the anonymity of participants, there will be limitations and conditions regarding the data which can be made accessible.

## Endnotes

^1^
We use the term ‘(intended) parent’ to refer to people who understood themselves as parents, regardless of legal status or biogenetic relationship, as well as those who had, or were still trying, to become parents in this way.
